# Expanding the phenotypic spectrum of *CLCN2*-related leucoencephalopathy and ataxia

**DOI:** 10.1093/braincomms/fcad273

**Published:** 2023-10-17

**Authors:** Paulo R Nóbrega, Anderson R. B. de Paiva, Katiane S Souza, Jorge Luiz B de Souza, Pedro Lucas G. S. B. Lima, Delson José da Silva, Milena Sales Pitombeira, Viviennee K Borges, Daniel A Dias, Luciana M Bispo, Carolina F Santos, Fernando Freua, Paulo Diego S Silva, Isabela S Alves, Leonardo B Portella, Paulina R Cunha, Rubens Paulo A Salomao, José Luiz Pedroso, Veridiana P Miyajima, Fábio Miyajima, Elisa Cali, Charles Wade, Annapurna Sudarsanam, Mary O’Driscoll, Tom Hayton, Orlando G P Barsottini, Stephan Klebe, Fernando Kok, Leandro Tavares Lucato, Henry Houlden, Christel Depienne, David S Lynch, Pedro Braga-Neto

**Affiliations:** Division of Neurology, Department of Clinical Medicine, Federal University of Ceara, Fortaleza, Ceara 60430-160, Brazil; Neurogenetics Unit, Department of Neurology, University of Sao Paulo School of Medicine, Sao Paulo, Sao Paulo 05403-000, Brazil; Neurogenetics Unit, Department of Neurology, University of Sao Paulo School of Medicine, Sao Paulo, Sao Paulo 05403-000, Brazil; Mendelics Genomic Analysis, Sao Paulo, Sao Paulo 02511-000, Brazil; Department of Neurology, São Rafael Hospital, Rede D’Or São Luiz, Salvador, Bahia 41253-190, Brazil; Neurogenetics Unit, Department of Neurology, University of Sao Paulo School of Medicine, Sao Paulo, Sao Paulo 05403-000, Brazil; Center of Health Science, State University of Ceara, Fortaleza, Ceara 3101-9795, Brazil; Faculty of Medicine, Federal University of Ceara, Fortaleza, Ceara 60430-160, Brazil; Universidade Federal de Goias, Goiania, Goias 74690-900, Brazil; Hospital Geral de Fortaleza, Fortaleza, Ceara 60150-160, Brazil; Department of Neurology, University of Sao Paulo School of Medicine, Sao Paulo, Sao Paulo 05403-000, Brazil; Hospital de Clínicas, Universidade Federal de Uberlândia, Uberlandia, Minas Gerais 38405-320, Brazil; Division of Radiology, Federal University of Ceara, Fortaleza, Ceara 60430-160, Brazil; Mendelics Genomic Analysis, Sao Paulo, Sao Paulo 02511-000, Brazil; University Hospital, EBSERH/Federal University of Sergipe, Aracaju, Sergipe 49060-676, Brazil; Universidade de Fortaleza, Fortaleza, Ceara 60811-905, Brazil; Hospital Infantil Albert Sabin, Fortaleza, Ceara 60410-794, Brazil; Neurogenetics Unit, Department of Neurology, University of Sao Paulo School of Medicine, Sao Paulo, Sao Paulo 05403-000, Brazil; Prevent Senior, Sao Paulo, Sao Paulo 01401-001, Brazil; Prevent Senior, Sao Paulo, Sao Paulo 01401-001, Brazil; Prevent Senior, Sao Paulo, Sao Paulo 01401-001, Brazil; Paris Brain Institute (ICM), Paris 75013, France; Ataxia Unit, Department of Neurology, Universidade Federal de São Paulo, Sao Paulo, Sao Paulo 04021-001, Brazil; Ataxia Unit, Department of Neurology, Universidade Federal de São Paulo, Sao Paulo, Sao Paulo 04021-001, Brazil; Centre for Clinical Diagnostics, Haematology and Haemotherapy Centre of Ceara (HEMOCE), Fortaleza, Ceara 60416-130, Brazil; Institute of Systems, Molecular and Integrative Biology, University of Liverpool, Liverpool L69 7BE, UK; Analytical Competence Molecular Epidemiology Lab (ACME), Oswaldo Cruz Foundation (Fiocruz), Fortaleza, Ceara 61773-270, Brazil; Postgraduate Program in Medical Sciences, Federal University of Ceará (UFC), Fortaleza, Ceara 60020-181, Brazil; Department of Neuromuscular Disease, UCL Queen Square Institute of Neurology, London WC1N 3BG, UK; Queen Square MS Centre, UCL Institute of Neurology, London WC1N 3BG, UK; Birmingham Children’s Hospital, Birmingham, Birmingham B4 6NH, UK; West Midlands Regional Clinical Genetics Service, Birmingham Health Partners, Birmingham Women’s Hospital NHS Foundation Trust, Birmingham B15 2TG, UK; University Hospital Birmingham, Birmingham B15 2GW, UK; Ataxia Unit, Department of Neurology, Universidade Federal de São Paulo, Sao Paulo, Sao Paulo 04021-001, Brazil; Department of Neurology, University of Würzburg, Essen 97080, Germany; Neurogenetics Unit, Department of Neurology, University of Sao Paulo School of Medicine, Sao Paulo, Sao Paulo 05403-000, Brazil; Mendelics Genomic Analysis, Sao Paulo, Sao Paulo 02511-000, Brazil; Neuroradiology Section, Hospital das Clínicas da Faculdade de Medicina da Universidade de São Paulo,Sao Paulo, Sao Paulo 05403-010, Brazil; Grupo Fleury, São Paulo, São Paulo 01333-011, Brazil; Department of Neuromuscular Disease, UCL Queen Square Institute of Neurology, London WC1N 3BG, UK; National Hospital for Neurology & Neurosurgery, London WC1N 3BG, UK; Institute of Human Genetics, University Hospital Essen, University Duisburg-Essen, Essen 45147, Germany; Department of Neuromuscular Disease, UCL Queen Square Institute of Neurology, London WC1N 3BG, UK; National Hospital for Neurology & Neurosurgery, London WC1N 3BG, UK; Division of Neurology, Department of Clinical Medicine, Federal University of Ceara, Fortaleza, Ceara 60430-160, Brazil; Center of Health Science, State University of Ceara, Fortaleza, Ceara 3101-9795, Brazil; Postgraduate Program in Medical Sciences, Federal University of Ceará (UFC), Fortaleza, Ceara 60020-181, Brazil

**Keywords:** ClC-2 chloride channels, leucoencephalopathies, MRI, ataxia

## Abstract

Mutations in *CLCN2* are a rare cause of autosomal recessive leucoencephalopathy with ataxia and specific imaging abnormalities. Very few cases have been reported to date. Here, we describe the clinical and imaging phenotype of 12 additional *CLCN2* patients and expand the known phenotypic spectrum of this disorder. Informed consent was obtained for all patients. Patients underwent either whole-exome sequencing or focused/panel-based sequencing to identify variants. Twelve patients with biallelic *CLCN2* variants are described. This includes three novel likely pathogenic missense variants. All patients demonstrated typical MRI changes, including hyperintensity on T_2_-weighted images in the posterior limbs of the internal capsules, midbrain cerebral peduncles, middle cerebellar peduncles and cerebral white matter. Clinical features included a variable combination of ataxia, headache, spasticity, seizures and other symptoms with a broad range of age of onset. This report is now the largest case series of patients with *CLCN2*-related leucoencephalopathy and reinforces the finding that, although the imaging appearance is uniform, the phenotypic expression of this disorder is highly heterogeneous. Our findings expand the phenotypic spectrum of *CLCN2*-related leucoencephalopathy by adding prominent seizures, severe spastic paraplegia and developmental delay.

## Teaser

Nobrega *et al*. describe 12 additional *CLCN2* leucoencephalopathy patients expanding the phenotypic spectrum by adding prominent seizures, severe spastic paraplegia and developmental delay. All patients demonstrated typical MRI changes. They found three novel missense variants. This report is now the largest case series of patients with *CLCN2-*related leucoencephalopathy.

## Graphical Abstract

**Graphical abstract:**
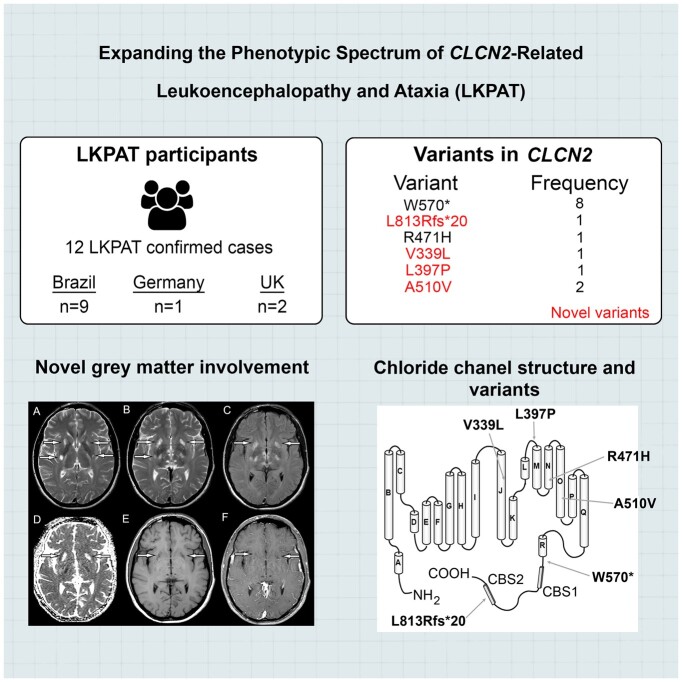

## Introduction

Biallelic pathogenic variants in *CLCN2* are associated with a rare autosomal recessive leucoencephalopathy with prominent ataxia^1^ recently named leucoencephalopathy with ataxia (LKPAT; Online Mendelian Inheritance in Man #615651). Since its first description in 2013, ∼31 individuals from 30 families have been reported.^1–10^ These observations led to the characterization of a typical phenotype with mild ataxia, variable cognitive impairment, visual impairment from chorioretinopathy or optic atrophy, male infertility and headache.^1^ Interestingly, all individuals reported to date have remained ambulatory, and current findings suggest very slow disease progression.

MRI reveals a typical pattern of T2 hyperintensity with variable restriction diffusion involving long white matter tracts, particularly the posterior limbs of the internal capsules, midbrain cerebral peduncles and middle cerebellar peduncles, caused by myelin oedema and vacuolization due to impairment in ClC-2 channel-mediated water and ion homeostasis. This imaging pattern is considered very suggestive of this disease.^1^

However, due to the very small number of cases reported, it is possible that very mild or atypical forms of this disease go unrecognized, leading to under-diagnosis. Furthermore, most cases of this disease have been observed in European and Asian populations, and few disease-causing variants have been reported so far.

In this study, we describe 12 additional patients with pathogenic or likely pathogenic variants in *CLCN2* including three novel missense variants as well as a recurrent nonsense variant associated with a possible founder effect in Brazil. The phenotypic spectrum in this series revealed previously unreported features including pure hereditary spastic paraplegia phenotype, prominent seizures, childhood developmental delay with autism spectrum disorder (ASD) and tremor. Characteristic changes on MRI typical of *CLCN2-*related leucoencephalopathy were seen in all patients.

## Materials and methods

Patients were identified through an active search for *CLCN2* pathogenic variants across the entire database of next-generation sequencing panels and whole-exome sequencing (WES) of a commercial laboratory (Mendelics Genomic Analysis, São Paulo, SP) and by contacting attending physicians to check for clinical and radiologic findings compatible with leucoencephalopathy (9 patients were obtained by this method). This database contains all patients investigated for numerous reasons, including a suspicion of genetic diseases, screening for cancer susceptibility, among other indications for WES. We also utilized network initiatives on neurogenetic diseases in Brazil, the UK (University College London) and Germany (University Hospital Essen) to search for additional patients with *CLCN2*-related leucoencephalopathy and ataxia (three patients were obtained by this method).

Informed consent was obtained from the patients in accordance with the Declaration of Helsinki, and the study was approved by the local ethical committee at Universidade Federal do Ceará under the number 5.952.628. DNA was extracted from oral swab samples, and WES was performed on a NovaSeq Illumina platform at Mendelics Genomic Analysis, São Paulo, SP, Brazil. Variants were categorized according to the American College of Medical Genetics guidelines.^11^ The following transcript was used as reference: NM_004366.6, ENST00000265593. Patients with biallelic pathogenic or probably pathogenic variants were recruited and attending physicians were contacted for clinical information. Clinical history, physical exam, MRI, and additional studies were reviewed for each patient.

Restricted diffusion on MRI was characterized by a visual analysis of lesions in diffusion-weighted image (DWI) and apparent diffusion coefficient (ADC) maps, as hyperintensity and hypointensity, respectively. The high signal on the DWIs suggested diffusion restriction, but unfortunately, we could not properly measure ADC values for all patients, and thus, a T2-shinethrough effect could not be entirely excluded.^12^

We reviewed the literature for articles in the English language reporting or researching LKPAT associated with *CLCN2* published between 1969 and 2023. The search terms ‘CLCN2’, ‘CLC-2 chloride channel’, ‘CL-2 CL-Channel’, ‘chloride channel clc-2’, ‘leucoencephalopathy’, ‘leucodystrophy’, ‘white matter disease’ and ‘lkpat’ were used to conduct the search in PubMed, Scopus, Embase, Online Mendelian Inheritance in Man and Cochrane databases. We also searched the reference lists of identified papers. The initial review was conducted in March 2023.

The search yielded 110 articles that were relevant, and 48 articles remained after duplicate identification. We added two articles from one of our references because of their significance to our discussion. After full-text reading, all of them were related to the scope of this review, with 15 case reports and 33 research papers regarding experimental models and molecular aspects of the disease.

Clinical, radiological and molecular data were extracted from reports and case series and used to characterize the clinical profile of LKPAT and compare with the findings of the present series. Molecular and experimental model studies were used to discuss radiologic and molecular findings in light of the most recent developments in the knowledge base of this disease.

### Statistical analysis

Data extracted from the participants were summarized using descriptive statistics. Categorical variables were expressed in absolute numbers and percentages. Continuous data were represented in mean values and standard deviation. Comparative statistical analyses were not performed.

## Results

We analysed 12 patients from 11 unrelated families (9 from Brazil, 2 from the UK and 1 from Germany), with age of onset varying between 6 months and 62 years (mean 25.95 years; SD 20.26), and among these 12, there were 7 males and 5 females. Most patients were adults (58%).

The most common clinical finding was ataxia in nine patients (75%), which varied from mild to severe cerebellar ataxia. The second most common finding was cognitive decline in five patients (42%), which ranged from cognitive developmental delay and regression to late-onset dementia. Most patients, however, were cognitively normal. Seizures were present in three patients (25%), which were mostly tonic–clonic seizures. Headache was reported by two patients. Prominent pyramidal signs were seen in two patients. In this series, we saw only one patient with self-reported visual impairment. An ophthalmological examination in this patient revealed signs of posterior and anterior uveitis. Visual-evoked potentials and/or digital retinal imaging were performed in two asymptomatic patients and were found to be normal in these patients.

Less common findings were myoclonus, transient ptosis, resting tremor, signs of ASD, psychosis, paroxysmal dyskinaesias and back pain, which were observed in only one patient each. One patient had hemiparesis attributed to a perinatal stroke. Hearing was inventoried clinically and none of the patients reported hearing abnormalities. Audiometry was not routinely performed. Consanguinity was reported in most patients.

**Figure 1:**
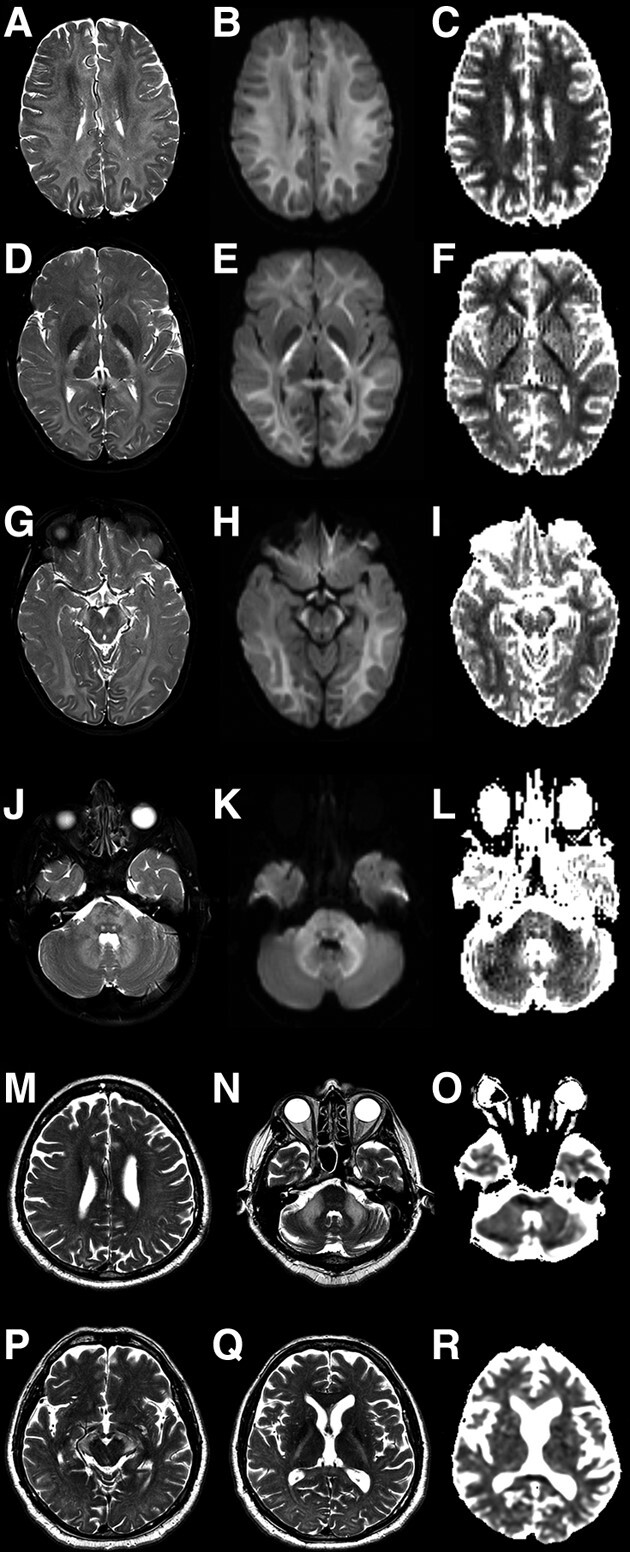
**MRI findings in patients with *CLCN2-*related leucoencephalopathy and ataxia.** Brain MR from patient 3 (**A**–**L**). Axial T2-weighted images (**A**, **D**, **G**, **J**) demonstrate symmetric bilateral hyperintensity involving the cerebral and cerebellar white matter, posterior limb of internal capsules, midbrain cerebral peduncles, central tegmental tracts (arrowheads in **G**) and middle cerebellar peduncles. The corresponding DWI (**B**, **E**, **H**, **K**) and ADC maps (**C**, **F**, **I**, **L**) suggest restricted diffusion, manifest as DWI hyperintensity and ADC map hypointensity, bilaterally in the subcortical cerebral white matter (arrows in **C**), posterior limb of internal capsules (arrows in **F**), midbrain cerebral peduncles (arrows in I) and middle cerebellar peduncles (arrows in **L**). Brain MR from patient 1 (**M**–**R**). Axial T2-weighted images (**M**, **N**, **P**, **Q**) show symmetric bilateral mild hyperintensity involving the cerebral white matter, which is more prominent in the subcortical region of the right parietal lobe (arrow in **M**); there is also hyperintensity in the posterior limb of internal capsules, midbrain cerebral peduncles, central tegmental tracts (arrowhead in **N**) and middle cerebellar peduncles. Also notice the compromise of the splenium of the corpus callosum (arrow in **Q**). The corresponding ADC maps (**O**, **R**) demonstrate hyperintensity in the posterior limb of internal capsules and middle cerebellar peduncles (arrows in **O**), which represents facilitation (not restriction) of the movement of water molecules. There is some restriction in the splenium of the corpus callosum, shown as hypointensity in the ADC map (arrowhead in **R**).

**Figure 2:**
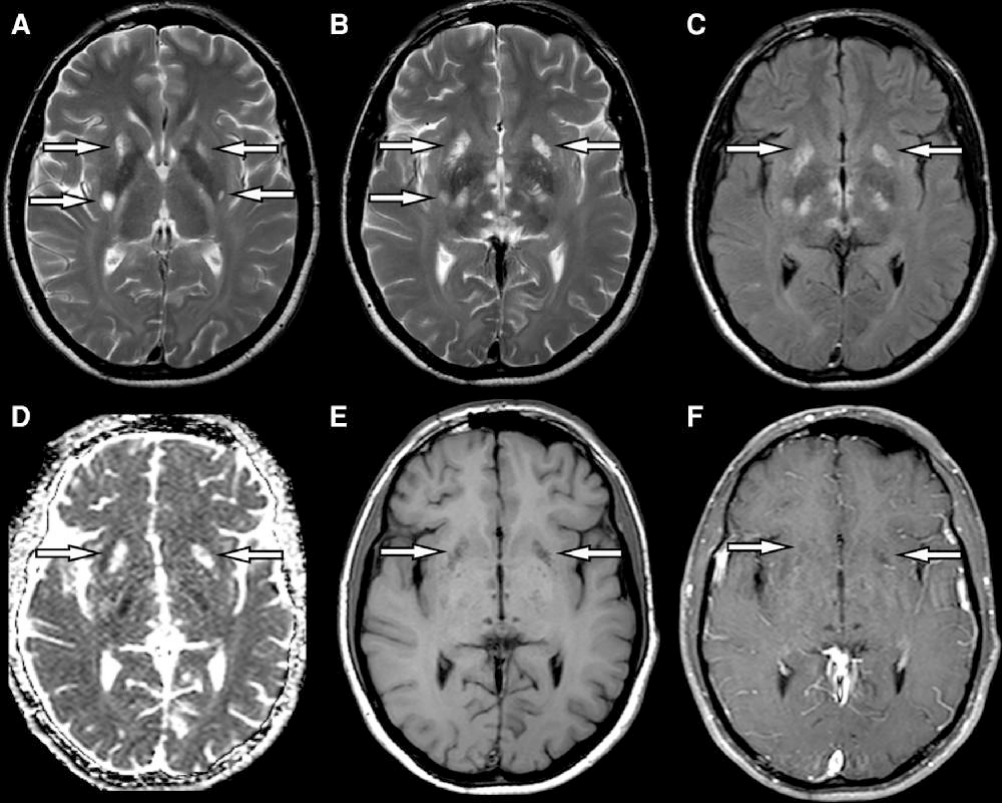
**Brain MR from patient 10 (A–F), demonstrating putaminal involvement.** Axial T2-weighted images (**A**, **B**) demonstrate hyperintense foci in the ventral and dorsal portions of both putamina (arrows). Axial FLAIR image (**C**) discloses the same hyperintensity (arrows). These foci present facilitated diffusion, characterized by hyperintensity in the corresponding ADC map (**D**, arrows). Axial T1 (**E**) and post-contrast T1 (**F**) demonstrate these foci as hypointense, without enhancement (arrows).

MRI revealed symmetric and bilateral T2-hyperintensity involving the posterior limbs of internal capsules, midbrain cerebral peduncles and middle cerebellar peduncles in all patients. Restricted diffusion was suggested by DWI and ADC maps in all 11 cases for which we had access to DWI images, ranging from mild DWI hyperintensity in the splenium of the corpus callosum to diffuse DWI hyperintensity of all long white matter tracts (Fig. 1). Diffusion restriction was more prominent in children than in adult patients. One patient (patient 9) had an area of encephalomalacia surrounded by gliosis in the right temporo-parietal region attributed to a perinatal stroke. In another patient, in addition to the white matter tract involvement previously described, there were also novel findings of grey matter involvement in the form of bilateral putaminal T_2_-hyperintensities with cavitation, involvement of the thalamus and caudate head (Fig. 2). Magnetic resonance findings are summarized in Supplementary Table 1.

**Figure 3:**
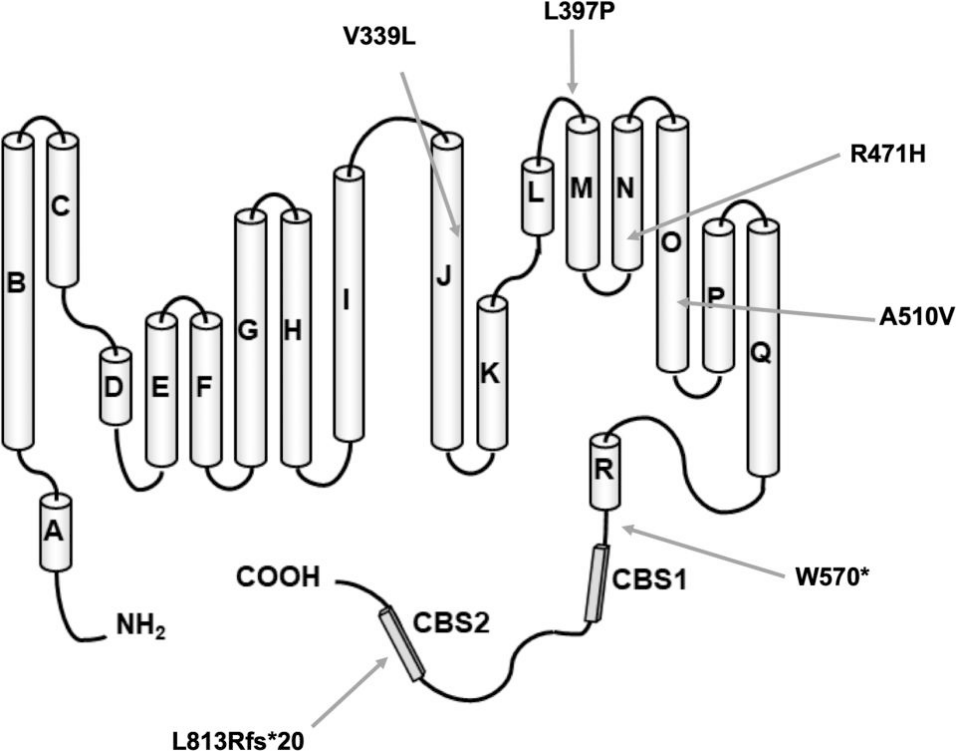
Schematics of the ClC-2 chloride channel showing protein domains and reported variants.

Most of the Brazilian patients (eight out of nine patients) had the previously reported c.1709G>A, p.Trp570Ter nonsense pathogenic variant in homozygosity or compound heterozygosity. One of the Brazilian patients had a novel frameshift likely pathogenic variant (p.Leu813Argfs*20) in compound heterozygosis with the c.1709G>A, p.Trp570Ter variant. One British patient had a previously reported missense pathogenic variant (c.1412G>A, p.Arg471His) in homozygosis.^5^ The other British patient had the novel likely pathogenic p.Leu397Pro variant in homozygosis. We identified three novel likely pathogenic missense variants: c.1015G>C, p.Val339Leu, c.1190T>C, p.Leu397Pro and c.1529C>T, p.Ala510Val (Fig. 3).

The p.Ala510Val variant was present in compound heterozygosis *in trans* with the c.1709G>A, p.Trp570Ter pathogenic variant in one case and in homozygosis in another. This novel variant has a gnomAD^13^ frequency of 0.0000443, with no homozygotes observed. It is predicted to be damaging by computational tools with a DANN^14^ score of 0.9993 and a CADD PHRED score of 24.8. It is a conserved residue with a GERP^15^ score of 5.1399. Alanine 510 is located in the O transmembrane helical domain. There are no established cut-off values for GERP scores in *CLCN2*, but a previous study analysing multiple genes found that all pathogenic variants had a GERP score of 2.95 or greater.^16^ According to the recommendations of the American College of Medical Genetics and Genomics,^11^ this variant was classified as likely pathogenic.

The c.1190T>C, p.Leu397Pro novel missense variant has a frequency on gnomAD^13^ of 0.00000398. Computational tools predict the variant to be damaging with a DANN^14^ score of 0.999 and a CADD PHRED score of 32. It alters a highly conserved residue with a GERP^15^ score of 5.73. It was present in one patient in homozygosis. According to the recommendations of the American College of Medical Genetics and Genomics,^11^ this variant was classified as likely pathogenic.

The c.1015G>C, p.Val339Leu novel missense variant was identified in the homozygous state in a female patient from Germany. It has a frequency of 0.00001064 (3 heterozygotes), with no homozygotes in gnomAD.^13^ This variant alters a conserved valine (up to zebrafish) of the J transmembrane domain of the channel and has a CADD PHRED score of 26.5. This variant was classified as of unknown significance but reclassified as likely pathogenic based on the highly concordant brain MRI findings suggestive of LKPAT.

The novel likely pathogenic p.Leu813Argfs*20 frameshift variant leads to a frameshift producing a stop codon. This variant is absent among 141 000 individuals in gnomAD,^13^ leads to loss of function, a known mechanism for this disease, and has been found in compound heterozygosis with the previously known p.Trp570Ter variant. It was, therefore, classified as likely pathogenic.

A summary of clinical findings and identified *CLCN2* variants with geographical origins may be found in Table 1. Individual case descriptions are available as Supplementary material.

**Table 1:** Summary of clinical features and identified variants in patients with *CLCN2-*related leucoencephalopathy and ataxia

Case	Sex	Age at onset	Presenting symptom	Clinical manifestations	Variants	Genotype	Origin
1	M	13	Spasticity	Spastic paraparesis	c.1709G>A,p.Trp570Ter	Homozygous	Brazil
2	F	30	Ptosis	Ptosis (unilateral, reversible)	c.1709G>A,p.Trp570Ter c.1529C>T, p.Ala510Val	Compound heterozygous	Brazil
3	M	8	Ataxia	Gait ataxia, dysarthria. Piramidal signs	c.1709G>A,p.Trp570Ter	Homozygous	Brazil
4	M	6 months	Developmental delay	Autism spectrum disorder, development delay, ptosis, ataxia	c.1529C>T, p.Ala510Val	Homozygous	Brazil
5	M	54	Fluctuating ataxia	Fluctuating ataxia, visual impairment	c.1709G>A,p.Trp570Ter	Homozygous	Brazil
6	F	18	Back pain	Back pain	c.1190T>C,p.Leu397Pro	Homozygous	United Kingdom
7	F	47	Seizures and headache	Seizures, headache, dysarthria, ataxia, myoclonus, memory impairment	c.1412G>A,p.Arg471His	Homozygous	United Kingdom
8	M	62	Behaviour changes, ataxia and seizures	Behaviour changes, cognitive impairment, ataxia, hyper-reflexia, seizures	c.2438delT,p.Leu813Argfs*20c.1709G>A,p.Trp570Ter	Compound heterozygous	Brazil
9	F	14	Headache	Headache, ataxia, seizures, cognitive decline, paroxismic dyskinaesia	c.1709G>A,p.Trp570Ter	Homozygous	Brazil
10	F	36	Psychosis	Psychosis, ataxia, postural and resting tremor	c.1015G>C, p.Val339Leu	Homozygous	Germany
11	M	3	Developmental delay, ataxia	Developmental delay, ataxia, strabismus	c.1709G>A,p.Trp570Ter	Homozygous	Brazil
12	M	26	Ataxia	Gait ataxia, action tremor, mild dysarthria	c.1709G>A,p.Trp570Ter	Homozygous	Brazil

## Discussion

*CLCN2* encodes the voltage-gated chloride channel CLC-2, which plays a role in brain ion and water homeostasis.^1^ Dysfunction of this chloride channel is hypothesized to lead to intramyelinic oedema, and therefore, the typical signal changes on MRI are observed, which characterize *CLCN2*-related leucoencephalopathy. Variability in phenotype has been reported since the first identification of this disorder, particularly regarding the age of onset.

ClC-2 channels are expressed in the brain mainly in the cell surface of fibrous astrocytes, particularly in the posterior limb of the internal capsules and dense white matter bundles, in astrocytic processes that run parallel or perpendicular to axonal bundles. However, a recent study using cell-type specific *CLCN2* knockout mice convincingly shows that loss of CLC-2 from both oligodendrocytes and astrocytes is required to generate a leucodystrophy phenotype (knockout in only astrocytes does not lead to white matter vacuolization),^17^ reinforcing the role of multiple glial cell types (the panglial syncytium) in ion and water homeostasis. There is also some expression inside axons and at the axonal surface contacting adaxonal myelin.^1^ In perivascular astrocytes, ClC-2 co-localizes with GlialCAM and MLC1. Cerebral white matter is formed mainly by myelinated axons that are involved in impulse conduction. Action potentials are conducted by neuronal depolarization mediated by sodium influx at the nodes of Ranvier, and the osmotic balance is maintained via potassium efflux in myelin-covered paranodal sites. The panglial syncytium is a network of astrocytes, oligodendrocytes and ependymal cells interconnected by gap junctions, which is essential for the disposal of ions and water after the osmolar shift induced by the action potentials. The astrocyte syncytium is particularly responsible for the buffering of rapid volume changes. ClC-2 appears to be involved in the generation of chloride currents necessary for the astrocytic regulatory volume decrease after action potential induced cell swelling, along with MLC1 (megalencephalic leucoencephalopathy with subcortical cysts associated with MLC1). Decreased ClC-2 function in the plasma membrane of astrocytes and olygodendrocytes leads to volume retention, myelinic oedema and vacuolation.^1^

Two other important proteins, MLC1 and GlialCAM, co-localize with ClC-2 in astrocytic endfeet at the perivascular basal lamina and seem to be part of the same water and ions homeostasis pathway. Both GlialCAM (also known as HEPACAM) and MLC1 are associated with megalencephalic leucoencephalopathy with subcortical cysts (MLC),^18,19^ another disease associated with myelin oedema and vacuolation. Different from *CLCN2*-associated LKPAT, in MLC, most compact white matter tracts such as the corpus callosum, internal capsule, brainstem and cerebellar tracts are spared. MLC1 and GlialCAM are both localized predominantly in distal astrocytic processes, while ClC-2 is more diffusely localized in astrocytes, and this might be related to the predominance of myelinic oedema in long white matter tracts in *CLCN2*.

Despite the fact that compact white matter tracts are spared in MLC, this disease is more severe than LKPAT. Subcortical white matter oedema in MLC is particularly pronounced, leading to early onset macrocephaly. There is a molecular interaction between ClC-2 and GlialCAM, but the disease caused by loss of function of ClC-2 or GlialCAM is very different, possibly due to a critical role of ion homeostasis in distal astrocytic processes predisposing to early white matter oedema.^20^

Depienne *et al*.^1^ found only 6 patients with *CLCN2* leucoencephalopathy out of a database of 3000 patients with undiagnosed leucoencephalopathy in Amsterdam. They hypothesize that either *CLCN2*-associated leucoencephalopathy is very rare, or it has a much wider phenotypic variation than previously thought.^1^ Our finding of nine new cases in Brazil in a database of ∼60 000 unselected exomes supports this second hypothesis, and screening radiology services for typical MRI findings might reveal an increasingly larger number of patients with very mild or even asymptomatic forms of this disease.

The lack of genotype–phenotype correlation in the small number of case reports to date is reinforced by this report. In this study, Patients 1, 3, 5, 9, 11 and 12 all carried the same homozygous nonsense mutation (p.Trp570Ter), yet there were significant differences not only in the age of onset but also in phenotypic expression between these patients. This variant was present in most cases from Brazil, possibly suggesting a founder effect, although no haplotype analysis was performed to determine ancestry, and therefore, this study is not sufficient to confirm a founder effect. There were 78 heterozygotes for this variant in the database from Mendelics Genomic Analysis, the commercial laboratory where we investigated all Brazilian patients.

The p.Trp570Ter is a loss-of-function variant previously described in the initial paper by Depienne *et al*.^1^, and this variant has been shown to lead to a downregulation of *CLCN2* mRNA and decreased expression of the resulting protein due to nonsense-mediated decay.^1^ Many variants described in this gene are loss-of-function variants, either nonsense or frameshift variants.^21^

We also identified one previously reported *CLCN2* missense variant (c.1412G>A, p.Arg471His), one novel likely pathogenic frameshift variant (p.Leu813Argfs*20) and three novel likely pathogenic missense variants, c.1015G>C, p.Val339Leu, c.1190T>C, p.Leu397Pro and c.1529C>T, p.Ala510Val. The novel likely pathogenic p.Leu813Argfs*20 frameshift variant leads to a frameshift producing a stop codon, similarly to other loss-of-function variants described in this gene.^21^

There are some previously described pathogenic missense variants in this gene. The mechanism by which some of these missense variants might lead to clinical phenotypes has been studied in more detail. These variants are predicted to affect the transmembrane helices B (p.Gly98Arg), N (p.Gly466Glu and p.Arg471His) and O (p.Ala500Val and p.Gly503Arg) of ClC-2 (Fig. 3). In a study of ClC-2 mutants expressed in Xenopus oocytes, none of the mutant ClC-2 channels produced by these variants have elicited activated chloride currents upon hyperpolarization when injected in *Xenopus* oocytes, and all mutants showed reduced plasma membrane expression, suggesting that the reduced ClC-2 function in these mutants is related to a severely impaired PM expression caused by abnormal trafficking.^22^

The novel p.Ala510Val missense variant is located in a codon close to the previously reported p.Ala500Val variant, which has been shown to produce CLC-2 channels that were restricted to the endoplasmic reticulum and scarcely expressed in the plasma membrane, possibly due to misfolding caused by the change of conserved hydrophobic amino acids in the transmembrane domains of ClC-2,^1^ which are also expected to be affected by the p.Ala510Val variant. Another study on the electrophysiological and biochemical properties of p.Ala500Val mutant ClC-2 channels indicated that this variant does not affect single-channel conductance, but instead changes the gating properties and expression of the channel at the plasmatic membrane, probably due to misfolding and impaired trafficking to plasma membranes.^21^ It is possible that a similar mechanism occurs with this novel p.Ala510Val variant, as well as with the previously described p.Gly503Arg missense variant.^22^

The most common clinical features of *CLCN2-*related leucoencephalopathy reported to date include cerebellar ataxia, visual impairment, headache and male infertility.^1^ Physical signs include ataxia, tremor, nystagmus and spasticity (Table 2). Uncommon presentations include early onset seizures^7^ and paroxysmal dyskinaesia.^3^

**Table 2:** Summary of clinical features and identified variants in previously published reports of CLCN2-related leucoencephalopathy and ataxia

Case	Sex	Age at onset	Presenting symptom	Clinical manifestations	Variants	Genotype	Origin
1	M	2 months	Seizures	Seizures; nystagmus; appendicular hypotonia; tremors	p.Glu690Ter	Homozygous	India
2	F	22 years	Headache	Cognitive impairment, ataxia, headache, spastic paraplegia, depressed mood	p.Trp570Ter	Homozygous	Tunisia
3	F	54 years	Tinnitus, vertigo	Ataxia, vision impairment, hearing loss, tinnitus, vertigo	N/R^a^	N/R^a^	North Africa
4	M	52 years	Asymptomatic	Asymptomatic	p.His590Pro	Homozygous	Morroco
5	M	36 years	Asymptomatic	Azoospermia	p.Gly503Arg	Homozygous	Italy
6	F	44 years	Action tremor, mild gait ataxia	Ataxia, tremor	p.Trp570Ter	Homozygous	North Africa
7	F	57 years	Tinnitus, vertigo	Ataxia, vision impairment, deafness, tinnitus, vertigo	p.Trp570Ter	Homozygous	North Africa
8	F	30 years	Vision impairment, psychosis	Ataxia, cognitive impairment, vision impairment, headache	p.Leu144_Ile145del	Homozygous	North Africa
9	F	12 years	Learning disability, headache	Spasticity, vision impairment, headache, ataxia, cognitive impairment	p.Gly382AlafsX34 p.Met22LeufsX5	Compound heterozygous	Europe
10	M	6 years	Headache	Ataxia, headache	p.Ala500Val	Homozygous	Europe
11	F	3 years	Action tremor, mild gait ataxia	Spasticity, vision impairment, ataxia, cognitive impairment, tremor	p.Arg277AlafsX23	Homozygous	Europe
12	F	21 years	Paroxysmal dyskinaesias	Ataxia, dyskinaesia, cognitive impairment	p.Ser375CysfsX6	Homozygous	Turkey
13	F	28 years	Gait difficulty, imbalance	Hearing loss, ataxia	p.Arg471His	Homozygous	Turkey
14	F	27 years	Headache, imbalance and blurry vision	Ataxia, headache	p.Glu475LysfsTer79	Homozygous	Turkey
15	F	46 years	Right-side numbness and ataxic gait	Ataxia	p.Leu435ArgfsTer7	Homozygous	Turkey
16	F	3 months	Tonic–clonic seizures	Seizures	p.Leu21Profs*27	Homozygous	Japan
17	F	22 years	Postural tremor in upper limbs	Cognitive impairment, tremor, vision impairment, tinnitus, dizziness	p.Arg753Ter	Homozygous	China
18	F	48 years	Ataxia	Ataxia	p.Gln385Ter	Homozygous	USA
19	M	11 years	Episodic headache, sensorineural hearing loss and vertigo	Headache, hearing loss, vertigo	p.P367L	Homozygous	India
20	M	6 years	Headache	Headache, hearing loss, vertigo	p.Leu21Profs*27	Homozygous	Japan

^a^N/R, not reported.

In addition to ataxia, we describe three patients with seizures as a prominent feature, a child with ASD and motor and speech delay, and late-onset patients with fluctuating ataxia and rapid deterioration in spastic paraplegia after injury. These findings might expand the phenotypic spectrum of *CLCN2*-related leucoencephalopathy and ataxia.

Before the identification of *CLCN2-*related leucoencephalopathy and ataxia, heterozygous variants in *CLCN2* were reported to be a cause of dominant idiopathic generalized epilepsy,^23–28^ but this finding was later refuted.^29,30^ Depienne *et al*.^1^ suggested that pathogenic variants in *CLCN2* were not associated with epilepsy after none of their six patients and heterozygous asymptomatic relatives had epilepsy. However, two patients with typical LKPAT and seizures were later described in the literature.^7,31^ In the present series, seizures were reported in one third of patients, suggesting that epilepsy may occur in association with biallelic pathogenic variants in *CLCN2*. The association of *CLCN2* heterozygous variants with epilepsy remains unproved so far.

We reported one patient with spastic paraplegia who became wheelchair-bound at the age of 50, after stabbing wounds to the chest and abdomen. To our knowledge, there are no previous reports of spastic paraplegia leading to loss of ambulation in *CLCN2*. Interestingly, this patient had no ataxia and no cognitive impairment, presenting as ‘pure’ hereditary spastic paraplegia. Worsening of white matter diseases after trauma has been previously reported in X-linked adrenoleucodystrophy^32^ and in vanishing white matter disease following trauma, systemic infections and even sunbathing.^33^ This deterioration has been attributed to pro-inflammatory cytokines and blood–brain barrier breakdown in XALD. It is not clear whether the trauma had any causal association with disease worsening in this patient, despite a clear temporal association.

Two siblings were previously reported with an overlap of SPG56- and *CLCN2*-associated leucoencephalopathy and ataxia, carrying biallelic pathogenic *CYP2U1* variants and also biallelic *CLCN2* likely pathogenic variants, with azoospermia and MRI compatible with *CLCN2*, suggesting a true overlap. Severe spastic paraplegia was attributed to SPG56 in these cases.^34,35^

Developmental delay with ASD has not been previously reported in LKPAT. A previous study on genetic risk factors for ASD has identified a single identical by descent alteration in *CLCN2*,^36^ but no LKPAT phenotype was reported in association with ASD. Despite the possibility that these psychiatric features are not related to *CLCN2* in these patients, the presence of co-occurring ataxia, typical MRI findings and the absence of other variants associated with ASD on WES for one patient suggest that ASD may be part of the LKPAT disease spectrum. Nevertheless, a description of additional cases with these features would be required to definitely conclude that they are part of *CLCN2*-related disorders.

Although we did not specifically search for male infertility, adult male subjects in this sample had no offspring (involuntary childlessness), suggesting possible infertility.

Despite the highly variable neurological phenotype, all patients had almost identical imaging abnormalities typical of *CLCN2*-related leucoencephalopathy. The striking DWI features involving long white matter tracts led us to suspect the diagnosis of *CLCN2* before WES results in most cases, demonstrating that this finding is a highly suggestive imaging sign.

*CLCN2*-related leucoencephalopathy should be suspected in patients presenting typical MRI findings, especially symmetric and bilateral T2-hyperintensity involving the posterior limbs of internal capsules, midbrain cerebral peduncles and middle cerebellar peduncles. Other structures commonly involved include the pyramidal tracts in the pons, the central tegmental tracts, cerebellar white matter, corpus callosum and cerebral white matter.^37^

Some peculiarities of imaging to be highlighted include the presence of restricted diffusion in some affected structures (Fig. 1), usually assigned to intramyelinic oedema caused by small vacuoles and extracellular spaces. The absence of restricted diffusion in most of the cerebral and cerebellar white matter can be related to the existence of large vacuoles and extracellular spaces in these locations, facilitating water movement.^1^ The reason for this difference in *CLCN2*-related leucoencephalopathy is not entirely understood. In one case, we identified a novel finding of grey matter involvement in the form of bilateral putaminal T_2_-hyperintensities with cavitation, caudate head and thalamic involvement (Fig. 2). It is not entirely clear whether grey matter involvement is associated with CLCN2 mutations in this case, but WES has been performed, which revealed no other variants of interest. There was no previous history of toxic exposure or other environmental factors.

In our cases, there was a trend towards a less prominent involvement of cerebral white matter in adult patients. As intramyelinic oedema can be reversible, there might be some reversal in the cerebral white matter changes with aging in these patients, in the same way that is observed in patients with megalencephalic leucoencephalopathy with subcortical cysts, caused by MLC1 and GlialCAM pathogenic variants that share similar mechanisms in water and ion homeostasis as explained previously. The first description by Depienne *et al*.^1^ also reported more prominent abnormalities in brain white matter among paediatric patients,^1^ further reinforcing this possibility of oedema reversal.

Another trend is to notice more widespread areas of restricted diffusion in younger patients (Fig. 1), which can also be explained by differences between small and large vacuoles, since at least in ClC-2 knockout mice, there is a progressive enlargement of the size of vacuoles with age.^38^ This means that in younger affected individuals, small vacuoles predominate, leading to diffusion restriction, while in older ones, there might be larger vacuoles in which diffusion facilitation can be appreciated.

We found no correlation between the extension of changes on MRI and the severity of clinical presentation.

This study has some limitations. Although it is the largest case series of *CLCN2*-related LKPAT to date, the number of patients is still too small to draw any genotype–phenotype correlations and to ascertain that some findings, such as ASD and psychosis, are definitely linked to the disease and are not a co-occurrence. Also, we did not perform functional studies to evaluate the biochemical effect of the novel variants reported. Nevertheless, we believe that this case series might draw more attention to the radiologic findings of this disease, leading to an increase in diagnostic suspicion and more cases being reported, especially in different populations from the original European and Asian populations.

This report expands the phenotypic spectrum of *CLCN2*-related leucoencephalopathy by adding prominent seizures, severe spastic paraplegia and developmental delay. These findings highlight the need to screen for *CLCN2* mutations when typical imaging features are present. Understanding the components of the ion and water homeostatic pathways might lead to strategies aimed at controlling or reversing myelinic oedema for the treatment of *CLCN2*-related leucoencephalopathy and related genetic diseases.

## Supplementary Material

fcad273_Supplementary_Data

## Data Availability

All data available are reported in the main manuscript or Supplementary material. Further data can be shared by the authors upon request.
