# Supplementary material for: Expanding the phenotypic spectrum of CLCN2-related leucoencephalopathy and ataxia
Source: Brain Commun. 2023 Oct 17;6(1):fcad273. doi: 10.1093/braincomms/fcad273 (PMC10763528; doi:10.1093/braincomms/fcad273)
Supplement: fcad273_Supplementary_Data [file fcad273_supplementary_data.pdf]

Patient 1

This Brazilian male patient developed stiffness of both legs, with pain and paraesthesia in the neck at the age of 13 years. One year later, lower limb strength rapidly deteriorated until he was unable to walk unaided and mobilised in a wheelchair. There was significant improvement in his condition with physical therapy and he became able to walk with unilateral assistance after 2 years. He was then well for most of his adult life, until the age of 50 years, when he was stabbed in the abdomen, after which he developed worsening weakness of both legs, only able to walk with bilateral assistance. The spinal cord (nor any other neural structure) was not damaged in the stabbing. There was no family history of neurologic diseases. His parents were consanguineous.

On neurological examination there was evidence of spastic gait, bilateral hyperreflexia and Babinski and Hoffmann signs, as well as impaired proprioception in lower limbs. There were no cerebellar signs or visual impairment. Cognition was normal, with an MMSE of 30/30.

Brain MRI showed hyperintensity on T_2-weighted images in the posterior limbs of the internal capsules, midbrain cerebral peduncles, middle cerebellar peduncles, pyramidal tracts in the pons, central tegmental tracts and splenium of the corpus callosum, as well as juxtacortical U-fibers. There was DWI hypersignal, more evident in the splenium (Figure 2).

WES identified a homozygous nonsense variant in *CLCN2* (NM_004366.6, c.1709G>A,p.Trp570Ter), previously described as pathogenic[1].

Patient 2

This Brazilian woman presented with acute left ptosis at the age of 30 and underwent a brain MRI scan. The ptosis spontaneously resolved after 3 days, and no additional neurological signs were observed. There was no history of headache, ataxia, or cognitive difficulty. Childhood development had been normal and there was no other medical history to note. She had no family history of neurological disorders, and her parents were non-consanguineous.

Brain MRI revealed symmetric white matter involvement. There was bilateral T_2-weighted hyperintensity in the posterior limbs of the internal capsules, midbrain cerebral peduncles, middle cerebellar peduncles, pyramidal tracts in the pons and central tegmental tracts, with DWI hypersignal in the cerebral peduncles and internal capsules. There was no abnormal enhancement. A 3x2mm saccular aneurysm of the ophthalmic segment of the left carotid artery was also noted.

Ophthalmic examination with computerized campimetry and digital retinal imaging was normal.

WES identified the previously reported pathogenic nonsense variant[1] in *CLCN2* (c.1709G>A,p.Trp570Ter), and a likely pathogenic novel missense variant, c.1529C>T, p.Ala510Val, *in trans*. The p.Ala510Val variant has a gnomAD[2] frequency of 0.0000443, with no homozygotes observed. It is predicted to be damaging by computational tools with a DANN[3] score of 0.9993. It is a conserved residue with a GERP[4] score of 5.1399.

Patient 3

This Brazilian boy developed slowly progressive gait ataxia and cerebellar dysarthria at the age of 8. Symptoms gradually worsened and by the age of 13 years, neurological examination revealed a broad-based ataxic gait with inability to tandem walk. Deep tendon reflexes were

diffusely brisk and there was mild cerebellar dysarthria. He had no family history of neurologic disease, and his parents were consanguineous.

Brain MRI disclosed symmetric white matter involvement suggestive of a genetic leukoencephalopathy. There was bilateral T_2-weighted hyperintensity in the cerebral white matter (diffuse), posterior limbs of the internal capsules, midbrain cerebral peduncles, middle cerebellar peduncles, pyramidal tracts in the pons and central tegmental tracts. Signal changes were also more diffuse in the pons and medulla, less prominent in the tegmentum, and in the deep white matter of the cerebellum. DWI hypersignal was observed in the subcortical white matter, posterior limbs of internal capsules, midbrain cerebral peduncles and middle cerebellar peduncles (Figure 2). There was no abnormal enhancement.

WES identified the same, previously described, homozygous pathogenic[1] nonsense variant in *CLCN2* found in patient 1(NM_004366.6, c.1709G>A, p.Trp570Ter).

Patient 4

This Brazilian boy was noted to have motor developmental delay at 6 months of age. He spoke a few words at 12 months, but then regressed and became non-verbal. He started walking with assistance at 20 months of age. At 2 years 8 months he was evaluated and was found to have poor visual contact and was unable to play symbolically – he received a diagnosis of autism spectrum disorder at this time. On neurological examination, at the age of 8, there was strabismus and mild bilateral ptosis, ataxic gait, normal deep tendon reflexes and limited speech. His parents were consanguineous. A paternal uncle had a diagnosis of cerebral palsy and epilepsy and lost the ability to walk at the age of 25, after an episode of pneumonia.

On Brain MRI there was bilateral T_2-weighted hyperintensity in the cerebral white matter, posterior limbs of the internal capsules, midbrain cerebral peduncles, middle cerebellar peduncles, pyramidal tracts in the pons, central tegmental tracts and in the deep white matter of the cerebellum. DWI hypersignal in the splenium of the corpus callosum, posterior limbs of internal capsules, midbrain cerebral peduncles and middle cerebellar peduncles. There was no abnormal enhancement.

WES identified the novel likely pathogenic homozygous missense variant in *CLCN2* (c.1529C>T, p.Ala510Val). This variant was also detected in patient 2.

Patient 5

This Brazilian man had a history of episodes of fluctuating ataxia, visual impairment and somnolence beginning at the age of 54 years. He also reported recurrent uveitis. There were no cognitive complaints. He had no previous history of neurologic disorders or rheumatologic conditions. Neurological examination revealed a broad based, ataxic gait and no other abnormalities. His family history was negative for neurologic diseases. His parents were consanguineous.

Brain MRI showed T_2-weighted hyperintensity in the posterior limbs of the internal capsules, midbrain cerebral peduncles, middle cerebellar peduncles, pyramidal tracts in the pons, central tegmental tracts and splenium of the corpus callosum, as well as juxtacortical U-fibres.

WES identified the homozygous pathogenic[1] nonsense variant in *CLCN2* (NM_004366.6, c.1709G>A, p.Trp570Ter), also identified in patients 1, 2 and 3.

Patient 6

This British woman of Pakistani heritage developed lower back pain at the age of 18, and was found to have loss of lumbar lordosis on MRI imaging. She was then investigated with further imaging of spine and brain, which identified the changes described below. The back pain resolved, and there were no other neurological symptoms or signs. Cognition was normal. Neurological examination was normal. She had no family history of neurologic disease, and her parents were consanguineous. Nerve conduction studies and visual evoked potentials were normal.

On Brain MRI there was bilateral T_2-weighted hyperintensity in the posterior limbs of the internal capsules, midbrain cerebral peduncles, middle cerebellar peduncles and cerebral white matter.

Focused exome sequencing identified a homozygous likely pathogenic novel missense variant in *CLCN2*, c.1190T>C, p.Leu397Pro. The variant frequency on gnomAD[2] is 0.00000398. Computational tools predict the variant to be damaging with a DANN[3] score of 0.999. It is a conserved residue with a GERP[4] score of 5.73.

Patient 7

This British woman of Pakistani heritage first developed symptoms in middle adolescence with seizures and headache. Her seizures have been in the form of absences and generalised tonic-clonic seizures, which have been relatively controlled on sodium valproate. In the third decade, she developed cerebellar dysarthria and some subtle memory impairment. Examination at the age of 47 demonstrated myoclonic jerks in the upper limbs and gait and appendicular cerebellar ataxia.

She had no family history of neurologic disease, and her parents were consanguineous.

On Brain MRI there was bilateral T_2-weighted hyperintensity in the posterior limbs of the internal capsules, midbrain cerebral peduncles, middle cerebellar peduncles and cerebral white matter.

A leukoencephalopathy NGS panel detected the previously reported[5] pathogenic *CLCN2,* c.1412G>A, p.Arg471His variant.

Patient 8

This Brazilian man first presented behavioural changes characterized by aggression and hypersexuality, in addition to cognitive impairment and gait disorder at the age of 71 years, followed by seizures. Neurologic examination at the age of 76 showed hyperreflexia in lower limbs, appendicular and gait ataxia and an MMSE of 16/30. There was no history of headache or visual impairment. He had no family history of neurologic diseases or consanguinity.

Brain MRI showed hyperintensity on T_2-weighted images in the posterior limbs of the internal capsules, midbrain cerebral peduncles, middle cerebellar peduncles, pyramidal tracts in the pons, central tegmental tracts, and splenium of the corpus callosum, as well as corona radiata bilaterally. There was no DWI hypersignalassociated.

WES identified a novel *CLCN2* pathogenic variant (p.Leu813Argfs*20) and the pathogenic nonsense variant (NM_004366.6, c.1709G>A,p.Trp570Ter), also found in patients 1,2,3 and 5.

Patient 9

This Brazilian woman presented with headache and gait abnormalities when she was 14 years old. Three years later, she developed cerebellar ataxia, seizures, cognitive decline, and paroxysmal dyskinesia. There was a history of depressive symptoms. She had a perinatal stroke with left hemiparesis sequelae. There was no consanguinity or family history of neurological diseases. Neurologic examination at the age of 25 showed diffuse hyperreflexia, spasticity, appendicular and gait ataxia and generalized dystonia.

On brain MRI there was bilateral T_2-weighted hyperintensity in the posterior limbs of the internal capsules, midbrain cerebral peduncles, middle cerebellar peduncles, pyramidal tracts in the pons, central tegmental tracts, and cerebral white matter. There was also an area of encephalomalacia surrounded by gliosis in the right temporo-parietal region.

WES identified a previously described homozygous pathogenic nonsense variant in *CLCN2* (NM_004366.6, c.1709G>A, p.Trp570Ter).

Patient 10

This German woman presented with psychosis at the age of 36. On clinical examination at age 40, she showed a broad-based gait, bilateral postural tremor and rest tremor on the left side that became obvious during walking. There were no other clinical signs of cerebellar or pyramidal involvement and cognition was normal.

On brain MRI bilateral T_2-weighted hyperintensity of the corpus callosum, internal capsules, cerebellar peduncles, pyramidal tracts, cerebral and cerebellar white matter was found. The EEG, nerve conduction studies and general laboratory parameters including CSF analysis were normal. A muscle biopsy at age 40 did not show any abnormalities. She had no family history of neurologic disease, and her parents were consanguineous.

A leukoencephalopathy NGS panel at age 40 detected a homozygous variant (c.1015G>C; p.Val339Leu) in *CLCN2.* This variant has a frequency of 0.00001064 (3 heterozygotes, 0 homozygotes) in gnomAD. This variant alters a conserved valine (up to zebrafish) of the J transmembrane domain of the channel and has a CADD PHRED score of 26.5. The re-evaluation of the clinical imaging data showed typical findings of LKPAT and led to reclassify the variant from VUS to likely pathogenic.

Patient 11

This Brazilian boy was noted to have motor developmental delay and ataxia at the age of 3. Ataxia showed a very slow progression over the years. At the age of 6 he was able to walk unassisted but was unable to perform tandem gait. He also has mild dysarthria. Convergent strabismus was present since early infancy. The patient did not acquire the ability to read or write and was diagnosed with moderate intellectual disability. His parents were consanguineous and family history revealed an older brother with slowly progressive ataxia.

Brain MRI revealed bilateral T_2-weighted hyperintensity in the posterior limbs of the internal capsules, middle cerebellar peduncles, cerebral white matter with mild T1 hypointensity and DWI Hypersignal in the internal capsules and middle cerebellar peduncles. There was also bilateral putaminal T_2-hyperintensities with cavitation; caudate head and thalamic involvement, pons and midbrain diffuse involvement sparing the substantia nigra and red nucleus.

WES identified a previously described homozygous pathogenic nonsense variant in *CLCN2* (NM_004366.6, c.1709G>A, p.Trp570Ter).

Patient 12

This Brazilian man, the older brother of patient 1, started with ataxia at the age of 26. He had no history of headache, visual impairment or seizures. Cognition was normal. He works as a truck driver and reports no disease progression over the last 3 years. His parents were consanguineous and family history revealed a younger brother with ataxia and developmental delay (patient 11). On neurological examination there was gait ataxia, action tremor and mild dysarthria.

Brain MRI revealed hyperintensity on T_2-weighted images in the posterior limbs of the internal capsules, midbrain cerebral peduncles, middle cerebellar peduncles, pyramidal tracts in the pons, central tegmental tracts, and splenium of the corpus callosum, as well as corona radiata bilaterally. There was DWI hypersignal in these areas.

Whole exome sequencing disclosed a previously described homozygous pathogenic nonsense variant in *CLCN2* (NM_004366.6, c.1709G>A, p.Trp570Ter), also found in patients 1,2,3,5, 8, 9 and 10.

References

1. Depienne C, Bugiani M, Dupuits C, et al. Brain white matter oedema due to ClC-2 chloride channel deficiency: An observational analytical study. *Lancet Neurol*. 2013;12(7):659-668. doi:10.1016/S1474-4422(13)70053-X

2. Lek M, Karczewski KJ, Minikel E V., et al. Analysis of protein-coding genetic variation in 60,706 humans. Nature. 2016;536(7616):285-291. doi:10.1038/NATURE19057

3. Quang D, Chen Y, Xie X. DANN: a deep learning approach for annotating the pathogenicity of genetic variants. Bioinformatics. 2015;31(5):761-763. doi:10.1093/BIOINFORMATICS/BTU703

4. Cooper GM, Stone EA, Asimenos G, Green ED, Batzoglou S, Sidow A. Distribution and intensity of constraint in mammalian genomic sequence. Genome Res. 2005;15(7):901-913. doi:10.1101/GR.3577405

5. Zeydan B, Uygunoglu U, Altintas A, et al. Identification of 3 Novel Patients with CLCN2-Related Leukoencephalopathy due to CLCN2 Mutations. Eur Neurol. 2017;78(3):125-127. doi:10.1159/000478089

Case	Age (MRI)	Internal capsules (posterior limb)	Cerebral peduncles	Middle cerebellar peduncles	Pyramidal tracts (pons)	Central tegmental tracts	Superior cerebellar peduncles	Decussation of SCP (midbrain)	Cerebellar white matter	Corpus callosum	Cerebral white matter	Restricted diffusion (DWI/ADC)
#1	56	+	+	+	+	+ (mild)	+	+	+	+ (mild, splenium)	+	+ (mild, splenium)
#2	31	+	+	+	+	+ (mild)	-	-	+	+ (mild, splenium)	-	+ (cerebr ped, int cap)
#3	9	+	+	+	+	+	+	-	+	+	+	+ (MCP, CTT, cerebr ped, int cap, CC - splenium too, subcortical WM)
#4	2	+	+	+	+	+	+	+	+	+	+	+ (subcortical WM, splenium, int cap, cerebr ped, MCP, cerebell WM)
#5	56	+	+	+	+	-	-	-	+	-	+	N/A
#6	17	+	+	+	+	+	+	+	+	+	+	+ (cerebr ped, splenium)
#7	44	+	+	+	+	+	+	+	+	+	+	+ (MCP, CTT, TCS pons, cerebr ped, int cap, splenium, cerebell WM)
#8	62	+	+	+	+	+	+	+	+	+ (mild, splenium)	+	+ (MCP, CTT, cerebr ped, int cap, splenium, corona radiata bilaterally, cerebell WM)
#9	18	+	+	+	+	+	-	-	+	+	+	N/A
#10	40	+	+	+	+ (mild)	+	+	-	+	+	+	mild ADC, DWI: "T2 shine through"
#11	25	+	+	+	+	+	+	+	+	+	+	+ (MCP, cerebr ped, int cap, splenium, cerebell WM)
#12	28	+	+	+	+	+	+	+	+	+	+	+ (MCP, CTT, TCS pons, cerebr ped, int cap, splenium, cerebell WM)

Case	Cerebral white matter involvement					
	**Subcortical**	**Deep**	**Periventricular**	**Perirolandic**	**Frontal pole**	**Other characteristics**
#1	+	+	-	+	+	Mild signal changes
#2	-	-	-	-	-	Mild changes CC splenium
#3	+	+	+	+	+	Diffuse (- genu CC)
#4	+	+	+	+	-	Patchy white matter changes
#5	+	-	-	+	-	N/A
#6	+	+	+	+	+	N/A
#7	+	+	-	+	+	N/A
#8	-	-	-	+	-	Small residual hemorrhage
#9	+	+	-	+	+	Encephalomalacia in the
#10	+	+	+	+	-	N/A
#11	+	+	+	+	-	Bilateral putaminal with
#12	+	+	+	+	-	N/A

Supplementary Table 1. Description of magnetic resonance findings in patients with CLCN2-Related Leukoencephalopathy and Ataxia.
